# Limited Weight Loss or Simply No Weight Gain following Lifestyle-Only Intervention Tends to Redistribute Body Fat, to Decrease Lipid Concentrations, and to Improve Parameters of Insulin Sensitivity in Obese Children

**DOI:** 10.1155/2011/241703

**Published:** 2011-03-14

**Authors:** Henry Marcano, Maricelia Fernández, Mariela Paoli, Mercedes Santomauro, Nolis Camacho, Rosanna Cichetti, Zarela Molina, Lenin Valeri, Roberto Lanes

**Affiliations:** 1 Unidad de Endocrinología Pediatrica, Hospital de Clinicas Caracas, Caracas, Venezuela; 2 Unidad de Endocrinología Pediatrica, Instituto Autónomo Hospital Universitario de Los Andes, Universidad de Los Andes, Mérida, Venezuela; 3 Unidad de Nutrición, Crecimiento y Desarrollo Infantil, Instituto Autónomo Hospital Universitario de Los Andes, Universidad de Los Andes, Mérida, Venezuela

## Abstract

*Objectives*. To investigate whether lifestyle-only intervention in obese children who maintain or lose a modest amount of weight redistributes parameters of body composition and reverses metabolic abnormalities. *Study Design*. Clinical, anthropometric, and metabolic parameters were assessed in 111 overweight or obese children (CA of 11.3 ± 2.8 years; 63 females and 48 males), during 8 months of lifestyle intervention. Patients maintained or lost weight (1–5%) (group A; n: 72) or gained weight (group B). *Results*. Group A patients presented with a decrease in systolic blood pressure (SBP) and diastolic blood pressure (DBP) ( and , resp.), BMI (), z-score BMI (), waist circumference (), fat mass (), LDL-C (), Tg/HDL-C ratio (), fasting and postprandial insulin (), and HOMA (), while HDL-C () and QUICKI increased (). Conversely, group B patients had an increase in BMI (), waist circumference (), SBP (), and in QUICKI (), while fat mass (), fasting insulin (), and HOMA () decreased. Lean mass, DBP, lipid concentrations, fasting and postprandial glucose, postprandial insulin, and ultrasensitive C-reactive protein (CRP) remained stable. *Conclusions*. Obese children who maintain or lose a modest amount of weight following lifestyle-only intervention tend to redistribute their body fat, decrease blood pressure and lipid levels, and to improve parameters of insulin sensitivity.

## 1. Introduction

The United States population has experienced a twofold to fourfold increase in the prevalence of obesity in the last two decades [1, 2], with a higher incidence among young African-Americans and Latinos [3]. Data obtained in a major city in the Venezuelan Andes seem to indicate a similar trend [4, 5]. Childhood obesity may lead to diabetes mellitus, cardiovascular disease, and premature death in adulthood [6]. Similarly, diseases such as dyslipidemias and type 2 diabetes are now being more frequently detected in both children and adolescents. Obese children have exhibited insulin resistance, elevated blood pressure, and increased concentrations of serum lipids. Higher body weight has been accompanied by an accumulation of visceral fat as well as elevated levels of alanine aminotransferase (ALT) and aspartate aminotransferase (AST) with hepatic steatosis [7–13]. Obese children have also been found to be in a proinflammatory state, characterized by increased ultrasensitive C-reactive protein (CRP) concentrations, changes in endothelial function, and alterations in arterial structure [14, 15].

The effect of modest nonpharmacological lifestyle-only intervention on auxological and metabolic parameters in obese children has not been conclusively determined, but several recent studies have demonstrated an improvement in body composition, a decrease in hemostatic and inflammatory markers, and an improvement in markers of insulin sensitivity following intervention [16–19]. It is not yet clear whether weight loss is required for these parameters to improve and to be sustained in time. In this study, we hypothesized that lifestyle-only intervention in obese children could lead to an improvement of both anthropometric and metabolic markers, even if weight loss was discrete or if weight remained stable.

## 2. Materials and Methods

### 2.1. Subjects

One hundred and eleven overweight or obese children (63 females and 48 males), with a mean chronological age of 11.3 ± 2.8 years, participated in the study. Fifty-three of the patients were prepubertal or in early puberty (Tanner stage I-II), whereas the rest were in puberty (Tanner stage III-IV). Patients originated from the Endocrinology, Nutrition, Growth, and Development Clinics of the Hospital de Clinicas Caracas in Caracas, Venezuela and the Instituto Autónomo Hospital Universitario de Los Andes (IAHULA) in Mérida, Venezuela. Only overweight or obese children, as determined by a BMI (kg/m^2^) above the 90th percentile or a Z-score BMI above 1.5 were included. Children with cardiac, endocrine, renal, and other chronic abnormalities and those receiving therapy (statins, glucocorticoids, omega 3, metformin, etc.) that could affect cardiovascular or metabolic function were excluded from the study. Patients with acute or chronic inflammatory disease that could affect inflammatory markers or those with physical impediments that could affect their exercise capacity were excluded from the study. All of our patients completed both baseline and 8-month auxological and laboratory evaluations, regardless of whether they followed lifestyle change recommendations.

### 2.2. Procedures

Patients were measured using a Harpenden stadiometer. Body mass index (BMI) was calculated, and subjects were classified utilizing the Fundacredesa-INN-USB's growth curves for Venezuelan children [20]. According to these curves the subjects were considered overweight if they had a BMI equal to or greater than the 90th percentile or a BMI z-score of >1.5. They were considered obese if their BMI was above the 97th percentile or if their BMI z-score was >2. The skin fold of the triceps was measured using a special caliper. Subsequently, measurements were taken of the circumferences of the left arm, the waist, and the hip using a measurement tape, as indicated by the National Health and Nutrition Examination Survey, 2000 [21]. The fat and lean mass were then calculated using a mathematical formula ( and , where  is fat mass,  is lean mass,  is the triceps fold measurement,  is the left arm circumference, and ) [20]. Blood pressure (BP) was measured in all children.

A fasting blood sample was obtained to determine levels of glucose, lipids, insulin, and CRP. Then, a glucose load of 1.75 grams per kilogram of body weight (maximum of 75 g) was administered orally to each patient. Two hours later, another blood sample was taken to determine glucose and insulin levels. Serum cortisol and thyroid function tests were measured at baseline in all patients and had to be normal to be included in the study. After the evaluation of baseline auxological and biochemical parameters, patients' weight, height, blood pressure, waist circumference, hip circumference, and arm circumference were measured monthly. Eight months later, the blood tests were repeated. Glucose, triglycerides (Tg), total cholesterol (TC), and high-density lipoprotein cholesterol (HDL-C) were measured by enzymatic methods with a Technicon autoanalyzer utilizing chemicals from Boehringer Mannheim Diagnostics. Low-density lipoprotein cholesterol (LDL-C) was calculated using the Friedwald formula (LDL-C = TC − Tg/5 + HDL-C) [22]. Insulin, cortisol, and thyroid function tests (TSH, FT4, and total T4) were measured by chemiluminescence utilizing an Immulite 1000 analyzer from Siemens Healthcare Diagnostics with interassay and intraassay coefficients of variation of 6.5% and 5.4%, 7.8% and 7.7%, 8.9% and 3.9%, 4.1% and 4.4%, and 6.7% and 6.7%, respectively. Ultrasensitive C-reactive protein (CRP) levels were determined by an immunometric assay (high-sensitivity enzyme immunoassay for the quantitative determination of C-reactive protein concentrations, DRG International, Inc, USA) with intraassay and interassay coefficients of variation of 7.5% and 4.1%. The insulin resistance index, HOMA-IR, was calculated with the following formula: Insulinemia (uU/mL) × glicemia (mmoL/L)/22.5. The insulin sensitivity index, QUICKI (quantitative insulin-sensitivity index), was calculated using the formula: 1/[(log insulin 0 min) + (log glicemia 0 min)] [23, 24]. This study was performed with the approval of the hospital's ethics committee, and authorization to participate in the study was obtained from parents and/or representatives.

### 2.3. Lifestyle Intervention Program

Patients and parents received a written form with general recommendations regarding nutrition and physical activity. Additionally, a subgroup was given a form in which to register weekly hours of physical activity, number of steps taken per day, and hours per week spent in sedentary activities, such as watching television or playing with a computer. In an initial meeting, children were informed of the changes in physical activity, behavior, and nutrition that were expected of them. Parents were stimulated to actively and responsibly participate in the program. 

Lifestyle changes included limiting the duration of television and computer activities. Patients were also encouraged to restrict their caloric intake by reducing the frequency of snack consumption, by exchanging high-calorie snacks for low-calorie, low-fat, low-carbohydrate snacks, and by limiting the consumption of sugar-based carbonated drinks and juices. A balanced diet (proteins, carbohydrates, and fat) with a high content of fiber and increased consumption of vegetables, grains, and fruits was recommended. Patients were stimulated to participate in the supervised sport activity of their choosing or, alternatively, to exercise at home on a treadmill or walk with their parents or older siblings. Initially, these activities would be performed three times weekly, but if possible their frequency would be increased to once per day. At the same time, the duration of the physical activity would be gradually increased in accordance with the child's exercise capacity. A subgroup of 36 patients were asked to determine the number of steps taken per day with the use of a pedometer (Omron model HJ-112INT), a device in the form of a watch that calculates the number of steps taken and the distance covered by the subject who carries it [25, 26]. We considered physical activity to be any regular sport, in addition to dance, aerobics, ballet, brisk walks, jogging, and school physical education. We quantified this activity in hours per week. In order to calculate the number of steps taken per day, the pedometer was used for 5 days. Then, the total number of steps taken during that period was divided by 5 to determine the average number of steps per day. This method was used to calculate the number of steps taken per day before initiating lifestyle-only intervention and once the intervention period was complete.

### 2.4. Statistical Analysis

The continuous variables are presented as mean ± standard deviation, and the categorical variables are shown in numbers and percentages. Initially the analysis was completed in all participants before and after intervention. Data was subsequently evaluated according to gender and pubertal development. Subjects were finally subdivided into 2 groups, one where they either maintained their weight or experienced a modest weight loss of 1–5% (group A; : 72, mean chronological age of 11.8 ± 2.7 years, 45 females and 27 males) and another where they experienced a mean weight gain of 6.6% (group B; : 39, mean chronological age of, 10.5 ± 2.7 years, 19 females and 20 males). The statistical differences of normally distributed variables were analyzed according to gender and pubertal status using the nonpaired Student's -test. The statistical differences of nonnormally distributed variables were analyzed according to gender and pubertal status using the Mann-Whitney test (blood pressure, insulin, HOMA-IR, and CRP). Furthermore, whereas the paired Student -test was used to analyze the statistical differences of normally distributed variables before and after intervention, the Wilcoxon test was employed to analyze those of nonnormally distributed variables. A logistic regression analysis to determine the influence of gender and pubertal development over the loss or gain of weight of patients was also performed. A  was considered significant. The statistical package SPSS, version 15, was utilized.

## 3. Results

### 3.1. Total Group

Children in our group as a whole had a significant decrease in BMI, z-score BMI, and fat mass () during the 8-month-long intervention period. While QUICKI increased () following intervention, LDL-C (), Tg/HDL-C ratio (), fasting insulin concentrations (), postprandial insulin concentrations (), and HOMA () decreased significantly. HDL-C levels were low at baseline and increased following intervention (). Waist circumference, waist/hip ratio, lean mass, SBP, DBP, fasting glucose, postprandial glucose, and CRP were normal at baseline and remained stable during the 8-month period. Results of the total group can be seen in Table 1.

**Table 1:** Laboratory and clinical variables before and after intervention of all obese children.

**Variables**	**Preintervention **	**Postintervention **
Height (cm)	148.14 ± 14.87	150.12 ± 14.06***
BMI (kg/m^2^)	27.22 ± 4.02	26.55 ± 4.06***
Z-score BMI	2.08 ± 0.34	1.90 ± 0.45***
Waist (cm)	85.65 ± 10.14	84.85 ± 10.23
Waist/Hip Ratio	0.89 ± 0.06	0.89 ± 0.07
Fat Mass (mm^2^)^a^	2760.3 ± 1049.1	2301.63 ± 664.8***
Lean Mass (mm^2^)^a^	3963.9 ± 1731.2	4171.0 ± 1229.2
SBP (mm Hg)	106.71 ± 12.52	106.61 ± 11.12
DBP (mm Hg)	70.59 ± 9.16	68.40 ± 8.29
Fasting Glucose (mg/dL)	82.33 ± 9.22	83.82 ± 9.22
2 Hr pl Glucose (mg/dL)	96.24 ± 17.89	94.05 ± 17.10
Fasting Insulin (mU/mL)	10.75 ± 8.49	8.19 ± 9.76**
2 Hr pl Insulin (mU/mL)	55.49 ± 53.23	40.03 ± 42.36**
HOMA	2.22 ± 1.80	1.63 ± 1.73***
QUICKI	0.36 ± 0.05	0.38 ± 0.04***
Tg (mg/dL)	119.74 ± 65.66	114.34 ± 54.85
TC (mg/dL)	168.52 ± 43.21	161.17 ± 31.80
HDL-C (mg/dL)	38.24 ± 9.99	43.37 ± 13.18*
LDL-C (mg/dL)	107.97 ± 39.49	95.94 ± 29.34**
Tg/HDL-C	3.26 ± 1.81	2.77 ± 1.46*
CRP (mg/dL)	0.24 ± 0.57	0.14 ± 0.13

^a^Calculated in 15 females and 21 males. *; **; ***.

We also analyzed the data of the total population according to gender (Table 2) and pubertal status (Table 3). Changes noted during the intervention period were mostly similar in males and females (both exhibited an increase in QUICKI levels and a decrease in BMI, z-score BMI, fat mass, fasting insulin, and HOMA. Neither showed changes in waist circumference, waist/hip ratio, lean mass, SBP, DBP, fasting glucose, postprandial glucose, TC, Tg, and CRP concentrations during this period). However, only females exhibited decreased postprandial insulin levels, a lower Tg/HDL-C ratio, and significant increases in HDL-C. Conversely, LDL-C concentrations decreased only in males (Table 2). Postprandial insulin levels, both before and after life-style only intervention, and postprandial glucose and fasting insulin concentrations before intervention were higher in females than in males. In contrast the waist/hip ratio, both before and after intervention, was increased in males (Table 2). Analysis of our data by pubertal staging revealed a similar decrease in BMI, z-score BMI, and fat mass and an increase in QUICKI in both prepubertal and pubertal children. No change in waist circumference, waist/hip ratio, lean mass, SBP, DBP fasting glucose, Tg, TC, HDL-C, Tg/HDL ratio, and CRP levels was noted in either group following intervention. Fasting insulin, postprandial insulin, HOMA, and LDL-C levels decreased significantly following intervention only in prepubertal children. Conversely, postprandial glucose levels decreased significantly only during puberty (Table 3). BMI, waist circumference, fat mass, lean mass, SBP, fasting and postprandial insulin, and HOMA were higher, and QUICKI was lower both before and after intervention in pubertal subjects (Table 3).

**Table 2:** Laboratory and clinical variables before and after intervention of obese children according to gender.

Variables	Female ()		Male ()	
	Preintervention	Postintervention	Preintervention	Postintervention

Height (cm)	148.46 ± 14.92	150.24 ± 13.62***	147.71 ± 14.95	149.96 ± 14.75***
BMI (kg/m^2^)	27.64 ± 4.71	26.88 ± 4.71*	26.68 ± 2.85	26.12 ± 3.01*
Z-score BMI	2.06 ± 0.37	1.87 ± 0.49***	2.11 ± 0.32	1.95 ± 0.40***
Waist (cm)	85.89 ± 11.82	84.68 ± 12.19	85.21 ± 7.37	84.61 ± 7.17
Waist/Hip Ratio	0.87 ± 0.07	0.87 ± 0.06	0.92 ± 0.05^††^	0.92 ± 0.05^††^
Fat Mass (mm^2^)^a^	2987.7 ± 1446.6	2362.0 ± 787.5*	2597.9 ± 628.2	2258.5 ± 578.5**
Lean Mass (mm^2^)^a^	3977.2 ± 2052.0	4272.6 ± 1416.3	3954.5 ± 1515.5	4098.3 ± 1107.6
SBP (mm Hg)	107.39 ± 12.51	108.00 ± 11.52	105.79 ± 12.90	106.54 ± 11.44
DBP (mm Hg)	69.93 ± 9.87	68.93 ± 8.37	70.14 ± 8.89	68.33 ± 9.05
Fasting Glucose (mg/dL)	83.59 ± 9.87	83.43 ± 9.43	80.86 ± 14.69	84.83 ± 9.61
2 Hr pl Glucose (mg/dL)	100.04 ± 19.89	95.59 ± 16.31	91.81 ± 17.06^†^	94.12 ± 18.98
Fasting Insulin (mU/mL)	12.59 ± 8.50	9.29 ± 6.49**	9.53 ± 9.06^†^	8.97 ± 12.68*
2 Hr pl Insulin (mU/mL)	71.72 ± 59.72	46.31 ± 29.26**	48.38 ± 50.79^††^	39.08 ± 54.09^††^
HOMA	2.66 ± 1.73	1.93 ± 1.43**	1.93 ± 1.95^††^	1.73 ± 2.14*
QUICKI	0.34 ± 0.04	0.37 ± 0.04***	0.37 ± 0.05^†^	0.39 ± 0.05*
Tg (mg/dL)	121.13 ± 66.00	104.00 ± 53.73	110.51 ± 57.15	121.76 ± 56.46
TC (mg/dL)	169.13 ± 42.53	160.60 ± 29.49	169.59 ± 47.09	158.93 ± 34.33
HDL-C (mg/dL)	40.06 ± 9.54	43.81 ± 9.03*	37.25 ± 9.37	41.69 ± 16.17
LDL-C (mg/dL)	105.49 ± 38.59	95.69 ± 25.54	113.49 ± 42.89	94.60 ± 31.99**
Tg/HDL-C	3.17 ± 1.76	2.39 ± 1.30*	3.36 ± 2.09	3.20 ± 2.09
CRP (mg/dL)	0.46 ± 0.80	0.15 ± 0.16	0.32 ± 0.70	0.42 ± 0.96

^a^Calculated in 15 females and 21 males. ^†^, ^††^ male preintervention versus female preintervention and male postintervention versus female postintervention; *, **, *** postintervention versus preintervention.

**Table 3:** Laboratory and clinical variables before and after intervention in prepubertal and pubertal obese children.

Variables	Prepuberty ()		Puberty ()	
	Preintervention	Postintervention	Preintervention	Postintervention

Height (cm)	137.87 ± 12.66	140.54 ± 12.13***	157.22 ± 10.10^†††^	158.61 ± 9.59^***†††^
BMI (kg/m^2^)	25.62 ± 3.42	24.83 ± 3.63*	28.54 ± 3.96^†††^	28.02 ± 3.85^*†††^
Z-score BMI	2.19 ± 0.31	1.98 ± 0.47***	1.96 ± 0.29^†††^	1.81 ± 0.37^***†^
Waist (cm)	80.75 ± 9.12	80.02 ± 8.53	90.79 ± 8.43^†††^	89.70 ± 9.83^†††^
Waist/Hip Ratio	0.92 ± 0.07	0.92 ± 0.07	0.87 ± 0.06^††^	0.87 ± 0.07^†††^
Fat Mass (mm^2^)^a^	2405.04 ± 753.91	2166.00 ± 624.89*	3684.20 ± 1178.32^††^	2654.30 ± 665.60^**†^
Lean Mass (mm^2^)^a^	3411.23 ± 1181.41	3706.65 ± 851.76	5401.10 ± 2144.89^†^	5378.30 ± 1272.13^††^
SBP (mm Hg)	101.59 ± 12.88	104.37 ± 11.96	111.00 ± 10.22^††^	109.98 ± 10.48^††^
DBP (mm Hg)	69.79 ± 10.63	68.79 ± 9.19	70.49 ± 7.99	68.45 ± 8.36
Fasting Glucose (mg/dL)	81.61 ± 9.51	84.09 ± 8.89	83.16 ± 14.41	84.14 ± 14.41
2 Hr pl Glucose (mg/dL)	92.35 ± 13.34	97.68 ± 16.78	100.19 ± 22.45^†^	92.41 ± 17.97*
Fasting Insulin (mU/mL)	9.28 ± 8.74	6.17 ± 5.97*	13.06 ± 8.73^†^	12.25 ± 11.49^††^
2 Hr pl Insulin (mU/mL)	55.95 ± 66.09	31.57 ± 32.40*	62.23 ± 47.19^†^	55.28 ± 47.03^††^
HOMA	1.85 ± 1.71	1.31 ± 1.38**	2.77 ± 1.91^††^	2.43 ± 1.91^†††^
QUICKI	0.37 ± 0.05	0.40 ± 0.05***	0.34 ± 0.03^†††^	0.35 ± 0.04^*†††^
Tg (mg/dL)	115.20 ± 65.65	111.85 ± 59.16	118.24 ± 59.14	112.38 ± 51.76
TC (mg/dL)	171.41 ± 40.49	161.66 ± 31.25	167.40 ± 48.63	158.61 ± 32.19
HDL-C (mg/dL)	38.85 ± 9.19	42.75 ± 12.38	38.70 ± 10.06	43.18 ± 13.22
LDL-C (mg/dL)	113.15 ± 34.78	96.48 ± 26.02*	105.21 ± 45.78	94.10 ± 31.43
Tg/HDL-C	3.12 ± 1.82	2.73 ± 1.62	3.41 ± 2.01	2.78 ± 1.53
CRP (mg/dL)	0.28 ± 0.68	0.13 ± 0.13	0.53 ± 0.82	0.55 ± 1.11

^a^Calculated in 26 Prepubertal and 10 Pubertal children. ^†^, ^††^, ^†††^ puberty preintervention versus prepuberty preintervention and puberty postintervention versus prepuberty postintervention; *, **, *** postintervention versus preintervention.

### 3.2. Group A

When we compared the variables before and after intervention, patients who either maintained their weight or had a modest weight loss presented with a significant decrease in SBP and DBP ( and ), in BMI, Z-score BMI, waist circumference (), and fat mass () following intervention. LDL-C (), Tg/HDL ratio (), fasting and postprandial insulin (), and HOMA () decreased significantly during the 8-month intervention period in these patients, while HDL () and QUICKI increased (). Fasting and postprandial glucose concentrations and CRP were normal at baseline and remained unchanged during the intervention period (see Table 4).

**Table 4:** Laboratory and clinical variables of group A (Lost Weight) and B (Gained Weight) children before and after intervention.

Variables	Group A ()		Group B ()	
	Preintervention	Postintervention	Preintervention	Postintervention

BMI (kg/m^2^)	27.91 ± 3.57	26.32 ± 3.87***	25.96 ± 4.52^†^	26.97 ± 4.40***
Z-score BMI	2.10 ± 0.36	1.82 ± 0.49***	2.05 ± 0.32	2.05 ± 0.35^†^
Waist (cm)	87.39 ± 9.00	84.75 ± 9.37***	82.18 ± 11.48^†^	85.08 ± 11.94**
Waist/Hip Ratio	0.89 ± 0.07	0.88 ± 0.06	0.89 ± 0.07	0.91 ± 0.05
Fat Mass (mm^2^)^a^	2915.41 ± 1326.75	2248.76 ± 727.70**	2621.63 ± 729.70	2348.94 ± 619.52*
Lean Mass (mm^2^)^a^	4126.06 ± 2221.60	4194.76 ± 1346.42	3818.95 ± 1180.42	4149.73 ± 1151.34
SBP (mm Hg)	111.02 ± 11.23	105.15 ± 10.51**	99.08 ± 11.11^††^	109.19 ± 11.85**
DBP (mm Hg)	70.57 ± 9.97	66.86 ± 7.15*	70.65 ± 7.71	71.12 ± 9.57^†^
Fasting Glucose (mg/dL)	82.35 ± 9.49	83.98 ± 10.68	82.30 ± 8.90	83.54 ± 8.01
2 Hr pl Glucose (mg/dL)	96.31 ± 19.51	92.19 ± 18.05	96.13 ± 15.03	97.29 ± 15.11
Fasting Insulin (mU/mL)	11.02 ± 6.78	7.48 ± 5.55**	10.29 ± 10.85	9.36 ± 14.25*
2 Hr pl Insulin (mU/mL)	55.04 ± 49.87	34.23 ± 26.73**	56.24 ± 59.64	49.93 ± 59.89
HOMA	2.27 ± 1.44	1.57 ± 1.28**	2.15 ± 2.31	1.75 ± 2.34*
QUICKI	0.35 ± 0.04	0.38 ± 0.04**	0.37 ± 0.05	0.39 ± 0.05**
Tg (mg/dL)	118.81 ± 55.78	105.30 ± 49.15	121.22 ± 80.07	128.75 ± 61.08
TC (mg/dL)	169.27 ± 46.08	158.22 ± 29.39	167.32 ± 38.94	165.92 ± 35.37
HDL-C (mg/dL)	38.75 ± 10.03	44.36 ± 13.19*	37.47 ± 10.07	41.84 ± 13.28
LDL-C (mg/dL)	109.82 ± 43.26	95.57 ± 29.24*	105.13 ± 33.47	96.50 ± 30.05
Tg/HDL-C	3.23 ± 1.53	2.51 ± 1.28*	3.31 ± 2.20	3.16 ± 1.65^†^
CRP (mg/dL)	0.18 ± 0.20	0.10 ± 0.08	0.31 ± 0.80	0.17 ± 0.16

^a^Calculated in 17 group A and 19 group B children. ^†^, ^††^ group B versus group A preintervention and postintervention. *, **, *** postintervention versus preintervention.

### 3.3. Group B

Patients who gained weight had a further increase in BMI (), waist circumference (), SBP (), and in QUICKI () during the 8-month intervention period. Fat mass (), fasting insulin (), and HOMA () decreased. Lean mass, DBP, lipid concentrations, fasting and postprandial glucose, postprandial insulin, and CRP remained stable during the study period. HDL-C levels were low at baseline and did not change during this period. Results of group B can be seen in Table 4.

When we compared group A with group B we noticed that, before intervention, BMI (), waist circumference (), and systolic BP () were significantly lower in group B (Table 4). After intervention, BMI Z-score (), diastolic BP (), and the Tg/HDL-C ratio () were significantly higher in group B.

In order to determine whether gender and pubertal development had a significant influence over the weight change during intervention, we performed a logistic regression analysis with weight gain or loss as a dependent variable. It demonstrated that the influence of gender () and pubertal development () was not significant.

### 3.4. Subgroup

In a subgroup of 36 patients, 16 who either maintained their weight or had a modest weight loss and 20 who gained weight, we analyzed the number of steps taken per day counted with a pedometer and the number of exercise hours per week. Patients in the former group lost weight and decreased their BMI, while children in the latter group increased their weight and BMI remained stable. The number of steps taken per day and the amount of exercise hours increased ( and , resp.) in both subgroups (Table 5). The number of hours spent watching TV or playing video games showed a tendency to decrease in patients who lost weight and of increasing in patients who gained weight. When we compared subgroups, we noticed that after intervention, BMI Z-score was significantly higher in patients who lost weight ().

**Table 5:** Anthropometric variables, calories, nutrients, steps taken per day, exercise in hours per week and hours per day spent watching TV and video games by obese children classified by their change in weight ().

Variables	Lost Weight (Group A) ()		Gained Weight (Group B) ()	
	Preintervention	Postintervention	Preintervention	Postintervention

BMI (kg/m^2^)	27,47 ± 25,05	25,04 ± 3,88**	27.02 ± 4.60	27.39 ± 4.97
Z-score BMI	2,12 ± 0,28	1,73 ± 0,52*	2.15 ± 0.28	2.14 ± 0.32^†^
Exercise (hs/wk)	2.37 ± 2.55	5.33 ± 3.89*	2.48 ± 2.48	5.10 ± 3.57*
Steps by Day	10070,00 ± 2931.65	23602.44 ± 4929.79**	9646.00 ± 2971.17	20507.33 ± 4193.69**
TV-Video (hs/day)	4.69 ± 2.98	4.05 ± 1.96	4.16 ± 1.93	4.29 ± 2.35

^†^ group B versus group A preintervention and postintervention. *, ** postintervention versus preintervention.

## 4. Discussion

Reports evaluating the effect of exercise on body fat have been contradictory, with some studies showing an increase in body fat but not in visceral fat, others demonstrating stable body composition, and yet others presenting a decrease in fat mass and an increase in lean body mass [17, 27, 28]. In our study, group A patients demonstrated an improvement in anthropometric parameters with a decrease in BMI, Z-score BMI, and waist circumference following moderate physical activity and diet-based lifestyle-only intervention. Additionally, in a subgroup of these patients, fat mass decreased significantly, and muscle mass remained stable following intervention. It is interesting to note how fat mass also decreased significantly in a number of group B patients, despite their weight increase. This suggests an important role for exercise in improving body composition, even in the absence of weight loss, as in this group of children an increase of physical activity as measured by the number of hours of exercise per week and by the number of steps taken was also noticed.

A proinflammatory state with an increase in CRP levels has been detected in obese children and is often accompanied by alterations in endothelial function and arterial structure [9, 14, 18, 29]. Studies of the effect of physical activity or lifestyle changes on inflammatory markers in children have shown mixed results. Whereas some report no effect on hemostatic or inflammatory markers following exercise or lifestyle-only intervention, others document a decrease in circulating fibrinogen concentrations [30–32] or a significant decrease in previously elevated levels of CRP, fibrinogen, and IL-6 [18]. In our study, a tendency towards a decrease in CRP levels following lifestyle-only intervention was found in all patient groups, but these levels remained in the normal range both before and after intervention. 

Recent studies have demonstrated the existence of a link between weight reduction in overweight children and improved insulin sensitivity following exercise or diet-based lifestyle-only intervention [16, 33]. In our study, fasting insulin levels and postprandial insulin levels decreased following intervention, while indexes of insulin resistance improved. These changes occurred even in the absence of weight loss or following only modest weight reduction. These findings are in agreement with recent reports in which short-term exercise-training programs were able to increase insulin sensitivity and cardiorespiratory fitness in obese children, even in the absence of weight loss and without measurable changes in body composition [17, 18]. However, whereas prepubertal children showed improvements in insulin resistance, children undergoing puberty did not. In the latter group, levels of fasting insulin, postprandial insulin, and HOMA were higher, and QUICKI values were lower. Compared to male patients, female patients were found to have lower QUICKI values and higher levels of fasting insulin, postprandial insulin, and HOMA. After intervention, both females and males had increased QUICKI values and lower levels of fasting insulin and HOMA. These findings demonstrate how insulin sensitivity can be different during the various stages of adolescence and between sexes. 

In our intervention study, concentrations of LDL cholesterol and the Tg/HDL-C ratio decreased, and HDL concentrations increased, both in the whole group and in group A children. Lipids remained stable in children who gained weight. Barter et al. [34] demonstrated how, in an obese group of adult patients, an increase in Tg and a decrease in HDL-C allowed for the identification of patients with generalized metabolic disorders. Similarly, in a recent study [4] we found that 69% of our obese children had an elevation of the Tg/HDL-C ratio that correlated with all markers of obesity and in a linear regression analysis proved to be, together with BMI, the main variable explaining the metabolic syndrome. 

We evaluated children of both sexes and included among them some that were in an age range in which puberty influences insulin resistance. Ideally, this study should have been performed separately in males and females using tighter age ranges. However, we did analyze our data according to gender (males versus females) and pubertal status (children in Tanner stages 1-2 versus children in Tanner stages 3–5). Another potential limitation of this study is the use of HOMA and QUICKI as measures of insulin resistance, instead of using the hyperinsulinemic-euglycemic clamp or the frequently sampled iv glucose tolerance test, which are considered to be more accurate. However, both HOMA and QUICKI have been validated as surrogate markers of insulin resistance in nondiabetic children and adolescents and compare quite well to the more sophisticated tests of insulin resistance [35, 36]. This study would have been strengthened by the use of a control group of nondieting and nonexercising obese children; however, it would have been very difficult and probably unethical for us to follow a subgroup of obese children without any form of intervention, considering that they sought our advise to lose excess weight and reduce accompanying comorbidities. 

In conclusion, a simple lifestyle-only intervention program, leading to either limited weight loss or no weight gain, helped redistribute body fat, decrease lipid levels, and improve parameters of insulin sensitivity in overweight and obese children. The reversibility of these auxological and metabolic abnormalities following moderate lifestyle-only intervention, even in the presence of very limited weight loss or simply no weight gain, has important implications for the future cardiovascular health of children and adolescents.
